# Bio‐Inspired Highly Brilliant Structural Colors and Derived Photonic Superstructures for Information Encryption and Fluorescence Enhancement

**DOI:** 10.1002/advs.202302240

**Published:** 2023-06-17

**Authors:** Xiaoru Liu, Junfu Liu, Boru Wei, Dongpeng Yang, Li Luo, Dekun Ma, Shaoming Huang

**Affiliations:** ^1^ School of Materials and Energy School of Physics and Optoelectric Engineering Guangzhou Key Laboratory of Low‐Dimensional Materials and Energy Storage Devices Guangdong University of Technology Guangzhou 510006 P. R. China; ^2^ Zhejiang Key Laboratory of Alternative Technologies for Fine Chemicals Process Shaoxing University Shaoxing 312000 P. R. China

**Keywords:** brilliant structural color, enhancing fluorescence, information encryption, photonic superstructures, refractive index contrast

## Abstract

Inspired by the brilliant and tunable structural colors based on the large refractive index contrast (Δ*n*) and non‐close‐packing structures of chameleon skins, ZnS–silica photonic crystals (PCs) with highly saturated and adjustable colors are fabricated. Due to the large Δ*n* and non‐close‐packing structure, ZnS–silica PCs show 1) intense reflectance (maximal: 90%), wide photonic bandgaps, and large peak areas, 2.6–7.6, 1.6, and 4.0 times higher than those of silica PCs, respectively; 2) tunable colors by simply adjusting the volume fraction of particles with the same size, more convenient than the conventional way of altering particle sizes; and 3) a relatively low threshold of PC's thickness (57 µm) possessing maximal reflectance compared to that (>200 µm) of the silica PCs. Benefiting from the core–shell structure of the particles, various derived photonic superstructures are fabricated by co‐assembling ZnS–silica and silica particles into PCs or by selectively etching silica or ZnS of ZnS–silica/silica and ZnS–silica PCs. A new information encryption technique is developed based on the unique reversible “disorder–order” switch of water‐responsive photonic superstructures. Additionally, ZnS–silica PCs are ideal candidates for enhancing fluorescence (approximately tenfold), approximately six times higher than that of silica PC.

## Graphical abstract

Chameleon‐inspired highly brilliant photonic crystals are prepared by non‐close–assembling high refractive index ZnS–silica core–shell structured particles in commercial acrylates. Furthermore, five derived photonic superstructures with distinct optical properties are fabricated by co‐assembly of ZnS–silica and silica particles, and particle‐selective etching strategy. These photonic superstructures show potential applications in information encryption and fluorescence enhancement.

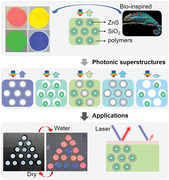

## 1 Introduction

**Figure 1:**
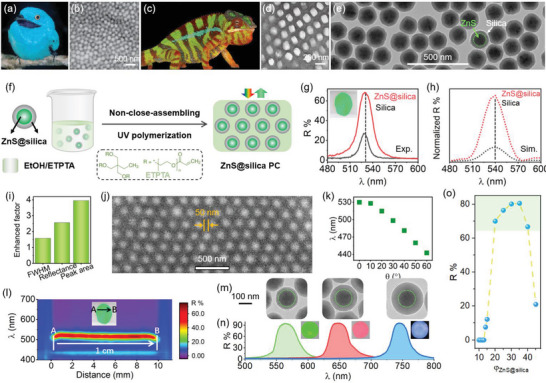
Fabrication and characterizations of ZnS–silica PCs. a) Male Plum‐throated Cotinga (*Cotinga maynana*, Cotingidae). b) Sphere‐type *β*‐keratin and air nanostructure from back contour feather barbs of *C. maynana*. c) Digital photo of a chameleon. d) SEM image of the skin of the Chameleon. e) TEM image of the ZnS–silica particles. The average size of ZnS–silica particle is 158 nm (core: 110 nm and shell: 24 nm). f) Schematic illustration of the fabrication of PC through the self‐assembly of the ZnS–silica particles. g,h) Reflection spectra of the silica and ZnS–silica PCs of g) experimental and h) simulated results. i) Enhanced factor of the FWHM, reflectance, and peak area of the PC compared to those of the PCS. j) SEM image and k) angle‐resolved spectra of the PC. l) Spatial reflection spectra of the ZnS–silica PC from the point A to B. m) TEM images of ZnS–silica particles (178, 212, and 248 nm) with the same ZnS (142 nm) core but different silica shell thicknesses (18, 35, and 53 nm) and the corresponding n) ZnS–silica PC with *φ*_ZnS–silica_ fixed to 30%. o) Reflectance of the ZnS–silica PC as a function of *φ*_ZnS–silica_. The thicknesses of all PCs are fixed to 57 µm. The images of the (a,b) bird and (c,d) chameleon were reprinted with permission from refs. [7, 8], respectively. a,b) REproduced with permission.^[^ ^7^ ^]^ Copyright 2009, Royal Society of Chemistry. c,d) Reproduced with permission.^[^ ^8^ ^]^ Copyright 2015 Springer Nature.

Structural colors,^[^ ^1^, ^2^, ^3^, ^4^, ^5^, ^6^ ^]^ existing in natural opals, butterfly wings, bird features, chameleons, etc., originate from the selective reflection of visible light by the periodically ordered structures with dielectric contrast. For instance, in the plum‐throated cotinga (*Cotinga maynana* [*C. maynana*]),^[^ ^7^ ^]^ the back feather barbs show non‐iridescent colors but faint turquoise‐blue color due to the nearly random close‐packed spherical air cavities (**Figure** **1**a,b). In striking contrast, chameleons^[^ ^8^ ^]^ show bright structural colors due to the strong coherent scattering of light caused by the large refractive index contrast (Δ*n*) between the non‐closely packed guanine nanocrystals and superficial iridophores (Figure 1c,d). In addition, the colors of chameleons can be altered through altering the distances between guanine nanocrystals due to the non‐close‐packing structures. Particularly, artificial chameleon skins^[^ ^9^, ^10^, ^11^, ^12^, ^13^ ^]^ based on the colloidal photonic crystals (PCs) have been extensively investigated because of their potential applications in displays,^[^ ^14^, ^15^, ^16^, ^17^, ^18^ ^]^ printings,^[^ ^19^, ^20^, ^21^, ^22^ ^]^ sensing,^[^ ^23^, ^24^, ^25^, ^26^, ^27^ ^]^ rewritable paper,^[^ ^28^, ^29^, ^30^ ^]^ anti‐counterfeiting,^[^ ^31^, ^32^, ^33^ ^]^ and optical devices.^[^ ^34^, ^35^, ^36^ ^]^

To achieve brilliant and tunable structural colors, large Δ*n* and non‐closely packed structures are necessary. In addition, the thicknesses and order degree require extra attention. Direct constructing non‐closely packed particles/polymer PCs with a large Δ*n* will be an ideal solution to fulfill the above requirements. Recent works proved that non‐close‐packing structures can be obtained by assembling silica particles in polymers.^[^ ^37^, ^38^, ^39^, ^40^ ^]^ However, the small Δ*n* (0.02–0.06) between the silica (*n*_silica_ = 1.46) particles and polymers (*n* = 1.44‐1.52) leads to the low reflectance (0–40%) and weak colors in most cases.^[^ ^13^, ^18^, ^32^, ^41^, ^42^ ^]^ Even worse, the reflectance of the silica PC decreases greatly (<10%) when its thickness is below 100 µm.^[^ ^26^ ^]^ Using particles with large *n* will be a promising and effective way to address this problem. So far, particles including Cu_2_O^[^ ^43^, ^44^ ^]^ (*n*_Cu2O_ = 2.7), CdS^[^ ^45^ ^]^ (*n*_CdS_ = 2.5), CeO_2_^[^ ^46^ ^]^ (*n*_CeO2_ = 2.20), Fe_3_O_4_^[^ ^16^, ^47^ ^]^ (*n*_Fe3O4_ = 2.42), and ZnS^[^ ^48^, ^49^ ^]^ (*n*_ZnS_ = 2.37) with high *n* have been prepared. Nevertheless, the inherent colors of Cu_2_O, CdS, CeO_2_, and Fe_3_O_4_ particles will affect the color purity and color saturation of PCs. Very recently, non‐close‐packed PCs^[^ ^49^ ^]^ with bright colors were prepared by etching the silica parts of the closely packed ZnS–silica/polymer composite PC with elaborate fabrications. Despite the large Δ*n* (0.4–0.6), the PC's reflectance (45%) is not as high as expected probably due to disturbance of the order degree by the etching processes. Therefore, it is still a big challenge to fabricate PCs with high reflectance, non‐closely packed ordered structures, brilliant and tunable colors with wide thicknesses.

In this work, inspired by the characteristics of chameleon skins and the previous works, PCs addressing the above drawbacks were fabricated by the direct non‐close‐assembling of ZnS–silica core–shell particles with high *n* in trimethylolpropane ethoxylate triacrylate (ETPTA) with a low *n* (Figure 1e,f). The ZnS–silica PCs show highly saturated colors with wide thicknesses due to the large Δ*n* (0.142–0.230) between the ZnS–silica particles and ETPTA (*n* = 1.470). The reflectance of PCs ranges at 70–98%, much higher than those of silica PCs (20‐40%) and conventional PCs. Benefiting from the non‐close‐packing structure, the colors of ZnS–silica PCs can be altered through adjusting the volume fraction of the particles with the same sizes, which is different from the traditional way of altering particle sizes. The silica shell is used to enhance the electrostatic repulsion between ZnS particles while retaining the high content of ZnS, which is crucial to obtain both non‐closely packed structures and bright colors. Based on the core–shell structure of the ZnS–silica particles and non‐closely packed structures of the PCs, a variety of new and complex derived PC superstructures can be obtained by co‐assembling the ZnS–silica and silica particles together into ZnS–silica/silica PC or by selective etching silica or ZnS of ZnS–silica/silica and silica PCs. Among these superstructures, HF‐etched ZnS–silica superstructures show unique water‐responsive “disorder–order” switch, giving rise to the reversible off–on colors under the dry and wetted state, respectively. Based on these characteristics, a new information encryption strategy was developed by simply combining these HF‐etched ZnS–silica superstructures with different optical performances. Moreover, it is shown that ZnS–silica PCs are excellent platforms which can enhance the fluorescent intensity by a factor of ≈10, approximately six times higher than that of silica PC. This work offers a simple, convenient, and robust strategy to fabricate highly brilliant PCs and derived advanced photonic structures and shows their potential application in fluorescence enhancement. These will upgrade the basic understanding of structural color and structures of PCs, and advance other applications such as displays, anti‐counterfeiting, optical devices, photocatalysis, and solar energy.

## 2 Results and Discussion

### 2.1 ZnS–Silica PC with Brilliant Color

Highly brilliant PCs were fabricated by 1) the preparation of ZnS–silica particles; 2) self‐assembling these particles in ETPTA to form long‐range ordered and non‐closely packed structures; and 3) fixing the ordered structures by photopolymerization (Figure 1e). Here, ZnS has a high *n*, which can efficiently improve the Δ*n* and thus reflectance of the PC. The silica shell coated on the surface of the ZnS particle is used to enhance the electrostatic repulsion between particles, thereby facilitating the self‐assembly of ZnS–silica particles in ETPTA. The small Δ*n* between silica (*n* = 1.460) and ETPTA (*n* = 1.470) can effectively reduce the incoherent scattering and thus enhance the color saturation of the PC. The usage of ETPTA can ensure a fast polymerization speed because each ETPTA molecular has three —CH=CH_2_ groups, therefore, the photopolymerization has little influence on the order degree of non‐close‐packing structures.

To prepare PC film with visible colors, uniform ZnS–silica particles (polydispersity index: 0.011) with an average diameter of 158 nm (Figure 1e, core: 110 nm and shell: 24 nm) were used. The transmission electron microscope (TEM) shows the nonuniform contrast of the ZnS because of its discontinuous crystal structure of the polycrystalline ZnS particle. Therefore, the *n* of the polycrystalline ZnS (1.910^[^ ^49^ ^]^) is similar to that of guanine crystal in chameleon skins but much lower than that of ZnS single crystals (*n* = 2.37). The *ζ*‐potential value of the ZnS–silica nanospheres (*ζ* = −43 mV in ethanol) is much larger than that of the original ZnS nanospheres (*ζ* = −7 mV in ethanol) so that the electrostatic repulsions between particles can be greatly enhanced. Therefore, the ZnS–silica particles could simultaneously possess good colloidal stability in solvents and a high *n* (*n* = 1.612) compared to the widely used silica (*n* = 1.460) particles. Briefly, these ZnS–silica particles were mixed with ethanol and ETPTA to form a uniform solution. After evaporation of ethanol, a liquid showing brilliant colors was obtained, indicating the ordered packing of ZnS–silica particles in ETPTA. This liquid was exposed to UV light to polymerize the ETPTA, thus fixing the ordered structures to obtain a solid and free‐standing PC film. The volume fraction of the ZnS–silica particles (*φ*_ZnS–silica_) and ETPTA (*φ*_E_) is 20% and 80%, respectively, implying the non‐close‐packing structures since the *φ*_ZnS–silica_ is far below that (74%) of the closely packed one. Without specific statement, the thicknesses of PCs are fixed to 57 µm.

The as‐fabricated ZnS–silica PC film possesses a highly brilliant green color with an intense reflection peak position located at 530 nm (Figure 1g). In comparison, silica PC (*φ*_silica_: 20%) based on a similar particle size (154 nm, Figure S1, Supporting Information) only shows weak green owing to its weak reflectance located at 526 nm. The reflection wavelength of the ZnS–silica PC is larger than that of the silica PC due to the larger *n* of ZnS–silica particles. These experimental results are similar to those of the results (Figure 1h) obtained by finite‐difference time‐domain (FDTD) simulations, suggesting the reflectance of PCs can be dramatically enhanced when ZnS–silica particles are used as the building blocks. The full width at half maximum (FWHM), reflectance, and reflection peak area of the ZnS–silica PC are 1.6, 2.6, and 4.0 higher than those of silica PC (Figure 1i), respectively, which can be explained by their different Δ*n*. Generally, the bandgap broadens, reflectance improves, and reflection peak area increases when Δ*n* increases.^[^ ^50^, ^51^, ^52^ ^]^ The Δ*n* of PCs can be calculated by Equation (1), where *n*_c_ and *n*_e_ are the *n* of the colloids and ETPTA, respectively. The average *n* of ZnS–silica particle (*n*_ZnS–silica_) can be calculated by Equation (2). *n*_ZnS_ and *n*_silica_ present the *n* of the ZnS core and silica shell, respectively. *D*_core_ and *D*_core–shell_ are the average diameter of the ZnS core and silica shell, respectively. The Δ*n* of the ZnS–silica PC is 0.142, 14.2 times higher than that of the silica PC (0.010), which greatly enhances the efficiency of light scattering at the interface of two dielectric materials and leads to a wider bandgap, higher reflectance, and larger peak area. Therefore, compared with silica PC, more saturated structural colors can be produced by the ZnS–silica PC which reflects more light into naked eyes.

### 2.2 Non‐Closely Packed Structure

Except for the brilliant color, the as‐fabricated PC has a non‐closely packed structure. As shown in scanning electron microscope (SEM, Figure 1j) top view image, ZnS–silica particles are non‐closely packed into face‐centered cubic structures with a long‐range order. The surface‐to‐surface distance between neighboring particles (*D*_s–s_) is measured to be 50 nm. In addition, the cross‐sectional SEM images (Figure S2, Supporting Information) of the central and rim regions of the same sample show similar results, demonstrating the good uniformity of non‐closely packed structures across the whole PC. Besides, the reflection wavelength of the PC blueshifts as the incident and detection angles increase simultaneously (Figure 1j), further verifying its long‐range ordered structures. The highly ordered structures can be attributed to the strong electrostatic repulsion between the particles.^[^ ^13^, ^53^ ^]^ Along with the evaporation of ethanol, the ZnS–silica colloidal solution concentrates and the average distance between the particles decreases accordingly. The particles begin to self‐assemble into ordered structures when the electrical double layers of particles start to overlap. A new balance was reached after evaporating almost all ethanol, leading to the highly ordered structures thanks to the strongly repulsive forces between the silica shells of ZnS–silica particles. In comparison, white solution was obtained when ZnS particles with poor electrostatic repulsions are used as the building block, proving the critical role of the silica shell in manipulation of the assembly behavior of ZnS particles.

The non‐closely packed structure also can be confirmed by reflection spectra. *D*_s–s_ can be calculated by Bragg's law (Equation (3)) and Equation (4), where *m* and *λ* are the diffraction order and reflection wavelength, respectively. *D*_id_ is the interparticle spacing between neighboring particles. *n* is the refractive index of the PC, and *θ* is the angle between the reflected beam and the normal. *n*_i_ and *φ*_i_ are the refractive indexes and volume fractions of each component of PCs. The *D*_s–s_ between neighboring particles can be calculated by Equation (5), where *D*_c_ is the diameter of colloidal particles. The *D*_s–s_ of the ZnS–silica PC is calculated to be 58 nm, consistent with the result obtained from the SEM image. The uniformity of structural color plays an important role for the applications of PCs. Here, we use spatial reflection spectra to characterize the uniformity of the PC by collecting the continuous reflection spectra from point A to B. As presented in Figure 1l, the small variation of the reflection wavelength and reflectance of the PC from point A to B suggest its good uniformity. These results demonstrated that the PC with the long‐range order, non‐closely packed structures, high reflectance, broad bandgap, and highly brilliant structural color can be fabricated based on the direct non‐close‐packing of ZnS–silica particles in ETPTA.

(1) $$\Delta n=n_{c}-n_{e}$$

(2) $$\begin{array}{ccc} n_{\mathrm{ZnS}-\mathrm{silica}} & = & n_{\mathrm{ZnS}}{\left(D_{\mathrm{core}}/D_{\mathrm{core}-\mathrm{shell}}\right)}^{3}\\ & & +n_{\mathrm{silica}}\left(1-{\left(D_{\mathrm{core}}/D_{\mathrm{core}-\mathrm{shell}}\right)}^{3}\right) \end{array}$$

(3) $$m\lambda =1.633\;D_{\mathrm{id}}\sqrt{n^{2}-\sin^{2}\theta }$$

(4) $$n^{2}=\sum n_{i}^{2}\varphi_{i}$$

(5) $$D_{s-s}=D_{\mathrm{id}}-D_{c}$$

Thanks to the high *n* of ZnS core, ZnS–silica particles with a wide range of shell thicknesses can be used for constructing highly reflective PCs. Here, by fixing the ZnS core to 142 nm, ZnS–silica particles (Figure 1m and Figure S3, Supporting Information) with silica shell of 18, 35, and 53 nm and corresponding *n* of 1.690, 1.595, and 1.545 were prepared. Correspondingly, these ZnS–silica particles possess *ζ*‐potential values of −40, −46, and −49 mV, implying the enhanced charge separation by a thicker silica shell. Then, PCs with *φ*_ZnS–silica_ of 30% were fabricated based on the non‐close–assembling strategy. Figure 1n and Figure S4, Supporting Information, show the high reflectance (≈90%), brilliant colors, and long‐range order of all these ZnS–silica PCs. Obviously, a thicker silica shell will induce a longer wavelength of the PC. It is surprising that these PCs show similar reflection intensity because ZnS–silica PC with a thinner silica shell is supposed to exhibit a higher reflectance due to its larger Δ*n*. This might be explained by the slight difference in *ζ*‐potential values. ZnS–silica particles with a large *ζ*‐potential value will assemble into more ordered structures, leading to a higher reflectance. Thus, the balance between the increase in reflectance by a large Δ*n* and the decrease in reflectance by a small *ζ*‐potential value induces the similar reflectance of these PCs. In this regard, the shell thickness has negligible effect on the reflectance and there is no optimized shell thickness (18–53 nm) for ZnS–silica particles. The change of ZnS‐particle size by altering shell thicknesses is more convenient than changing the size of ZnS core in practical applications.

In addition to Δ*n*, the *φ*_ZnS–silica_ is also a crucial parameter to the reflectance. For non‐close–assembling, there is a threshold of *φ*_ZnS–silica_ (13%, Figure 1o), over which ZnS–silica particles start to assemble into ordered structure. The ZnS–silica PC can be divided into: crystal regions with ZnS–silica particles packed into ordered structures and amorphous regions with particles randomly moved due to Brown motion. When the *φ*_ZnS–silica_ is below 13%, no colloidal crystals were formed, resulting in a flat reflectance. When the *φ*_ZnS–silica_ is slightly larger than 13%, only a small number of particles assemble into crystals and the density of crystal region is low, leading to a weak reflectance. As *φ*_ZnS–silica_ gradually increases to 35%, more and more particles participate into crystal region; therefore, reflectance increases accordingly. ZnS–silica particles become too crowded for ordered packing after further increasing *φ*_ZnS–silica_ (40–45%), thereby leading to the decrease in reflectance. In addition, no structural color but white powders can be obtained when *φ*_ZnS–silica_ exceeds 45%. Thus, *φ*_ZnS–silica_ of 20–40% should be the good choice for achieving PCs with high reflectance.

Compared to other ZnS–silica PCs, the decrease in the reflectance of ZnS–silica PC with the *φ*_ZnS–silica_ of (40–45)% can be attributed to the decrease in order degree since the refractive index contrast is the same for all samples. The order degree depends on the electrostatic repulsions between ZnS–silica particles and ionic strength. A strong electrostatic repulsion and low ionic strength are favorable for a high order degree and intense reflectance. For ZnS–silica PCs, with a *φ*_ZnS–silica_ < 35%, the increase in *φ*_ZnS–silica_ decreases the interparticle distance (*D*_id_) of ZnS–silica particles, leading to enhanced electrostatic repulsions and thus the increased tendency in reflectance. In contrast, the increase in *φ*_ZnS–silica_ will cause the increase in ionic strength, leading to the decreased tendency in reflectance. As the former one dominates the order degree, as a result, the reflectance increases slightly along with the increase in *φ*_ZnS–silica_. However, when *φ*_ZnS–silica_ > 35%, the further increase in ionic strength will decrease the electrostatic repulsions because the electrical double layers of ZnS–silica particles were significantly compressed by the absorption of increased counterions, causing the decrease in reflectance.

It should be noted that no coffee ring was observed for the ZnS–silica PC by the non‐close–assembling strategy. The coffee‐ring effect usually originates from the selective deposition of colloids on the outmost layer of the colloid/solvent solution when conventional self‐assembly methods, such as drop‐casting and dip‐coating, are used. For these approaches, it is difficult to avoid the coffee ring effect and achieve excellent uniformity since the formation and fixation of ordered structures occur almost simultaneously, both of which depend on solvent species, substrates, temperature, and humidity. In this work, the colloidal/solvent solution consists of silica/ethanol/ETPTA. After selective evaporation of ethanol, silica particles were uniformly packed in ETPTA into a long‐range order which was then fixed by UV polymerization. Unlike the traditional ways, the formation and fixation of uniform structures can be independently and efficiently controlled, thus avoiding the coffee ring effect. Additionally, the gaps between silica particles fulfilled by ETPTA also can prevent unfavorable cracks compared to conventional PCs.

### 2.3 Tunable Structural Colors

**Figure 2:**
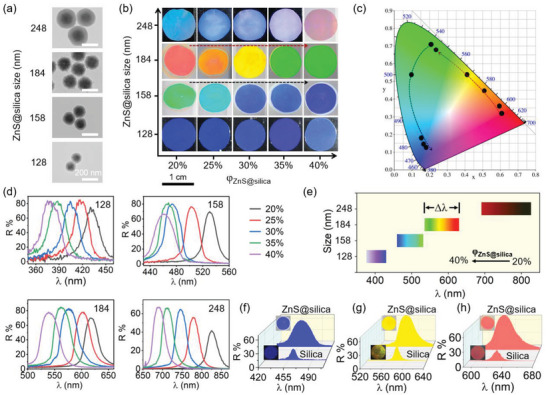
Tunable structural colors by altering *φ*_ZnS–silica_ and ZnS–silica particle sizes. a) TEM images of ZnS–silica particles with different size of 128 (core: 92 nm and shell: 18 nm), 158 (core: 110 nm and shell: 24 nm), 184 (core: 142 nm and shell: 21 nm), and 248 nm (core: 142 nm and shell: 53 nm). b) Digital photos of ZnS–silica PCs with different *φ*_ZnS–silica_ (20‐40%) and with particle sizes of 128–248 nm. The diameter of each sample is 1 cm. c) CIE diagram of the PCs with the ZnS–silica particle size of 158 and 184 nm. d) Reflection spectra of the ZnS–silica PCs with different *φ*_ZnS–silica_ (20–40%) and with particle sizes of 128–248 nm. e) Reflection wavelength tuning range of the ZnS–silica PCs relative to the size ZnS–silica particles. f–h) Reflection spectra of the ZnS–silica and silica PCs. The size of ZnS–silica particle in (f), (g), and (h) is 158 (*φ*_ZnS–silica_: 40%), 184 (*φ*_ZnS–silica_: 30%), and 184 nm (*φ*_ZnS–silica_: 20%), respectively. The size of silica particle in (f), (g), and (h) is 160 (*φ*_silica_: 40%), 186 (*φ*_silica_: 30%), and 186 nm (*φ*_silica_: 20%), respectively. The thicknesses of all PCs are 57 µm.

Different from the closely packed PCs requiring tens of particle sizes to tune the structural colors, almost all visible colors of ZnS–silica PCs can be obtained by simply altering the *φ*_ZnS–silica_ based on limited particle sizes. Here, four ZnS–silica particles with sizes ranging from 128 to 248 nm (**Figure** **2**a and Figure S5, Supporting Information) were used as candidates for fabricating PCs. As shown in Figure 2b, for the same *φ*_ZnS–silica,_ the structural color red shifts as the particle size increases due to the increase of *D*_id_ (Figure S6, Supporting Information). For the same particle size, the structural color blue shifts as the *φ*_ZnS–silica_ increases owing to the decrease in *D*_id_ (Figure S7, Supporting Information). According to Bragg's law, the increase or decrease in *D*_id_ will cause the redshift or blueshift of wavelengths and structural colors, respectively. Compared to other PCs, 158 and 184 nm ZnS–silica PCs show highly brilliant colors, covering most of visible colors. To evaluate the color saturation of these PCs, their reflection spectra are converted into the black points in the CIE chromaticity diagram (Figure 2c). The hue of the PC can be identified directly through observing the color where the black point locates at. The color saturation is high and weak when the black point locates near the edge and center, respectively. The CIE diagram shows that all these black points are close to the edges, verifying the highly saturated structural colors of these PCs in human eyes.

To determine the color tuning range of the PC by varying *φ*_ZnS–silica_, their reflection spectra were collected. Figure 2d shows that most PCs show high reflectance (60–81%) corresponding to the brilliant colors due to their large Δ*n*. The slight difference in reflectance can be attributed to the difference in order degree. In addition, the change in reflection wavelength as the function of *φ*_ZnS–silica_ is well consistent with the variation of colors. Taking the 184 nm ZnS–silica PC as the example, its reflection blueshift from 619 to 538 nm with a wavelength tuning range (Δ*λ*) of 81 nm when *φ*_ZnS–silica_ increases from 20 to 40%, in good agreement with the color change from red to green. The peak variation of ZnS–silica PCs due to the change of *φ*_ZnS–silica_ or particle size is consistent with the calculated results (Tables S1 and S2, Supporting Information). Correspondingly, the transmission (Figure S8a, Supporting Information) of these PCs is almost zero and not very high when the wavelength is shorter and longer than stopbands, respectively. This can be ascribed to the incoherent scattering of light by the large Δ*n* that enhances the light scattering efficiency and strong absorption of ZnS–silica particles at short‐wavelength regions (Figure S8b, Supporting Information).

The Δ*λ* of ZnS–silica PCs depends on the particle sizes. The Δ*λ* increases from 54 to 135 nm (Figure 2e) when ZnS–silica particle increases from 128 to 248 nm accordingly. This can be attributed to the gradual increase in *D*_s–s_ (from 47 to 92 nm accordingly). A larger *D*_s–s_ is more favorable for a large shift of wavelength when increasing *φ*_ZnS–silica_, thus, leading to a larger Δ*λ*. The FWHM, reflectance, and peak area of the typical blue, yellow, and red ZnS–silica PCs are 2.8–3.6, 2.7–3.5, and 6.5–8.8 times higher than those of silica PCs (Figure 2f–h) with similar particle sizes (Figure S9, Supporting Information). It is worth noting that highly brilliant yellow (size: 184 nm and *φ*_ZnS–silica_: 30%) has been obtained despite its broad peak profile, which is a big challenge for silica PCs. Therefore, compared to the conventional way, the non‐close–assembling strategy can efficiently and conveniently achieve most visible colors with only two different sizes, which will facilitate their practical applications.

Except these, one may find that 248 nm ZnS–silica PCs with *φ*_ZnS–silica_ of 20, 25, and 30% show dim green, lake blue, and blue colors, probably due to the Mie scattering of these ZnS–silica particles. The reflection peak position of Mie scattering can be roughly calculated by *λ*_Μ_ = *n*_c_ *D*_id_, where *λ*_Μ_ is resonant wavelength. Here, the *λ*_Μ_ of the 20%, 25%, and 30% samples is calculated to be 525, 494, and 472 nm, respectively, in good agreement with their colors. Unfortunately, no obvious resonant reflective peaks can be detected. Such phenomenon requires extra investigations and efforts to reveal the inherent mechanism.

### 2.4 Optimized Thickness (*T*)

The reflectance of PCs is not only dependent on the Δ*n* but also positively related to the periodic number of ordered structures. For PCs, there is a threshold of the thickness (*T*_th_), with which the PCs possess the maximal reflectance. For *T* < *T*_th_, the increase in *T* will lead to more efficient coherent and incoherent scattering of light by the increased periodic structures and increased defects, respectively. At this stage, the incoherent scattering of light is not strongly enough to influence the color visibility of PCs. Therefore, the reflectance increases when the *T* increases. For *T* > *T*_th_, despite the increase of *T*, the reflectance will not increase but the incoherent scattering increases due to the further increase of the number of defects, causing the pale and whitish colors of PCs.

**Figure 3:**
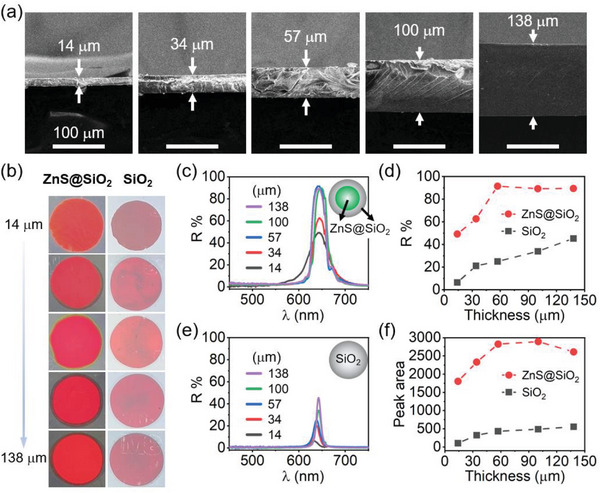
Effect of thicknesses. a) Cross‐sectional SEM images of ZnS–silica PCs with different thicknesses. b) Digital photos of ZnS–silica and silica PCs with different thicknesses. c,d) Reflection spectra of c) ZnS–silica PCs and d) silica (213 nm) PCs. e) Reflectance and f) Peak areas of the PCs as a function of thickness. ZnS‐SiO_2_: 210 nm (core: 120 nm and shell: 45 nm).

To investigate the influence of *T* on the reflectance, ZnS–silica particles with the size of 242 nm (core: 120 nm and shell: 45 nm, Figure S10, Supporting Information) were used to fabricate the PCs with diverse thicknesses. As shown in **Figure** **3**a, a wide range of thicknesses (14–138 µm) can be easily achieved by simply altering the thickness of the interval using the non‐close–assembling approach, which is exceedingly difficult for conventional assembly methods. For all samples, *φ*_ZnS–silica_ is fixed to 35%. For comparison, silica PCs were also fabricated by replacing the ZnS–silica particles with silica particles (Figure 3b, *φ*_silica_ fixed to 35%). Compared to silica PCs, ZnS–silica PCs show much brilliant red colors (Figure 3b) and more saturated colors (Figure S11, Supporting Information) with a wide range of thicknesses. For ZnS–silica PCs, their reflectance and peak areas first increase and then maintain nearly constant once the *T* increases over 57 µm (Figure 3c,d), suggesting the *T*_th_ of the PC is or near 57 µm. However, the case is quite different for silica PCs whose reflectance and peak areas increase gradually when *T* increases, indicating the *T*_th_ of the silica PC is larger than 138 µm. According to the previous work,^[^ ^54^ ^]^ the *T*_th_ of the silica PC might be located at around 270–360 µm. The striking differences in *T*_th_ between the ZnS–silica and silica PCs can be attributed to their different efficiency in coherent scattering of light, which is proportional to the Δ*n*. For silica PCs, their Δ*n* are small, leading to weak reflectance, narrow bandgaps, and the low efficiency in reflecting light. For ZnS–silica PCs, their Δ*n* are large, resulting in intense reflectance, wide bandgaps, and high efficiency of coherent scattering of light. Therefore, compared to silica PCs, light can be reflected more efficiently by ZnS–silica PCs with the same thickness, which induces the small *T*_th_. It is worth noting that the ZnS–silica PC still shows high reflectance (≈50%) when the PC is as thin as 14 µm, which is 7.6 times higher than that of silica PC with the same thickness. Additionally, the saturation thickness of the ZnS–silica PC is similar (≈57 µm, Figures S12–S14, Supporting Information) regardless of the ZnS core diameter, silica shell thickness, and ZnS–silica particle volume fraction.

One may find that the FWHM of the 14 µm ZnS–silica PC is much larger than others. For a PC, its FWHM depends on the order degree and refractive index contrast (Δ*n*). A higher order degree and a smaller Δ*n* will be favorable for a small FWHM. For ZnS–silica PCs, their Δ*n* is constant, therefore, it is reasonable to infer that the large FWHM of the 14 µm ZnS–silica PC might be attributed to the decrease in order degree. It should be noted that the shoulder peaks at 565 nm originate from the spectrometer and will be amplified when overlapping with reflection wavelengths (Figure S15, Supporting Information) due to the light modulation effect by photonic bandgaps. Overall, the high reflectance of the PC with a broad thickness will be useful in some applications such as optical devices and smart windows.

### 2.5 Derived Photonic Superstructures

**Figure 4:**
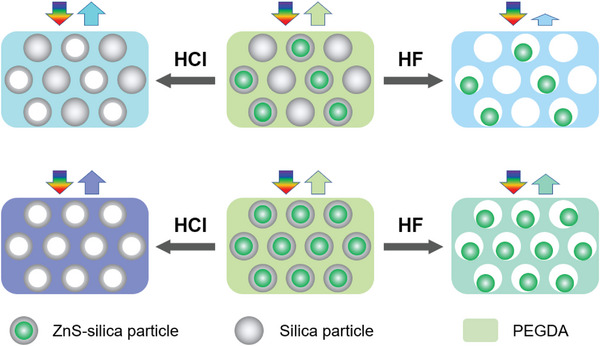
Schematic illustration of the fabrication of derived photonic superstructures. In this figure, the size of ZnS–silica particle is 172 nm (ZnS core: 142 nm and shell: 15 nm) and the size of silica particle is 180 nm.

Thanks to the core–shell structures of ZnS–silica particles and the non‐close‐packing structure, a variety of new photonic superstructures (**Figure** **4**) can be fabricated through 1) co‐assemble ZnS–silica and silica particles into ZnS–silica/silica PCs, and 2) selective etching the ZnS or silica from ZnS–silica/silica PCs and ZnS–silica PCs. Here, 172 nm ZnS–silica particles (core of 142 nm and shell of 15 nm, Figure S16, Supporting Information) and silica particles (180 nm) with similar sizes are used to fabricate ZnS–silica/silica PC through self‐assembly strategy. A thin silica shell is favorable for etching. *φ* of particles is 30% with *φ*_ZnS–silica_:*φ*_silica_ = 1:1 so that nearly equal numbers of ZnS–silica and silica particles is introduced into PCs. ETPTA is a hydrophobic polymer, which hinders the etching. Therefore, the hydrophilic poly(ethylene glycol) diacrylate (PEGDA, Figure S17, Supporting Information) is selected to replace ETPTA to fabricate PCs.

**Figure 5:**
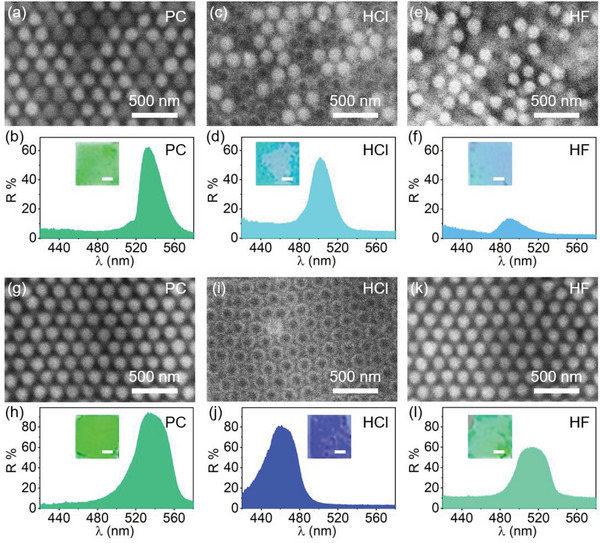
Photonic superstructures. a,c,e) SEM images and b,d,f) reflection spectra and photos of the a,b) ZnS–silica/silica PC, c,d) corresponding HCl etched PC, and e,f) HF etched PC. g,i,k) SEM images and h,j,l) reflection spectra and photos of the g,h) ZnS–silica PC, i,j) corresponding HCl etched PC, and k,l) HF etched PC. The scale bar in photos is 1 mm. In this figure, the size of ZnS–silica particle is 172 nm (ZnS core: 142 nm and shell: 15 nm) and the size of silica particle is 180 nm. The thicknesses of all PCs are fixed to 57 µm.

Under SEM (**Figure** **5**a and Figure S18, Supporting Information), ZnS–silica particles with high brightness and silica particles with low brightness are alternatively packed into long‐range ordered and non‐closely packed structures similar to that of ZnS–silica PC. This ZnS–silica/silica PC features a green color and a strong photonic bandgap at 533 nm (Figure 5b). Although there are two Δ*n* in the film, *n* is a certain value for the PC according to Equation (4); therefore, ZnS–silica/silica PC shows a single green color and a certain reflection wavelength according to Bragg's law. After etching by HCl, ZnS was removed selectively from ZnS–silica particles, leaving silica particles with hollow structures. Thus, HCl etched PC (Figure 5c) with solid silica and hollow silica particles alternatively packed can be observed. Compared to the pristine PC, the reflection wavelength of the HCl etched PC blueshifts nearly 30 nm with a lake blue (Figure 5d) due to decrease in *n*. Noticeably, its reflectance is still intense, because the ordered structure is still retained after etching. In contrast, a different derived photonic superstructure was obtained when the silica was selectively etched by HF. As shown in Figure 5e, silica particles and the silica shells of ZnS–silica particles are removed away, leading to the break of long‐range order and only some short‐range order can be observed. Thus, the HF etched PC exhibits a weak reflectance and neglectable color (Figure 5f). Despite the low reflectance, its reflection wavelength is still angle‐dependent (Figure S19, Supporting Information). However, the reflectance decreases nearly to 0 when the incident and detection angles are large than 20° simultaneously, suggesting a decrease in the angle‐dependence. Similarly, compared to the pristine PC, the blueshift of the reflection peak position also can be attributed to the decrease of *n*.

Additional two derived PCs also have been fabricated through selective etching the core or the shell of ZnS–silica PC. As shown in Figure 5g, ZnS–silica particles are non‐closely packed, possessing a reflection signal located at 534 nm and a typical green color (Figure 5h). After etching by HCl, all ZnS were removed and hollow silica particles were non‐closely packed (Figure 5i) in etched PC, causing a large blueshift of reflection wavelength and a blue color (Figure 5j). When the ZnS–silica PC was etched by HF, a new derived superstructure (Figure 5k) can be generated. However, it is hard to compare the *D*_s‐s_ before and after etching due to the difficulty in recognizing the thickness of the silica shell. The etching of silica shells from ZnS–silica particles can be confirmed by the variation of the reflection signal (Figure 5l). The replacement of silica shell by air causes the slight blueshift of reflection wavelength, while the decrease in Δ*n* induces the decrease of reflectance. As the order degree was retained, the reflectance of the photonic superstructure is still intense. Its reflection wavelength is angle‐dependent (Figure S20, Supporting Information), further proving the ordered structure after etched by HF.

ZnS–silica particles are non‐closely packed with polymers filled between neighboring particles, resulting in a dramatic decrease in the resolution of SEM. Therefore, no obvious voids can be observed from the SEM images of the HF‐etched samples. ZnS–silica PCs with thicker silica shells will be favorable to show the voids. Here, the ZnS–silica (core of 142 nm and shell of 35 nm, Figure S21a, Supporting Information) PC was fabricated, which possesses highly ordered structures (Figure S21b, Supporting Information) and high reflectance at 640 nm (Figure S21c, Supporting Information). After etching in HF, the reflectance of the PC decreases dramatically to almost zero due to the break of order degree and the voids can be clearly seen (yellow arrows, Figure S21d, Supporting Information). This HF‐etched PC, named water‐responsive photonic superstructure, was then immersed in water, interestingly, an intense reflection peak located at 681 nm appears, suggesting the recovery of order degree due to the move of ZnS particles. Compared to the pristine PC, its wavelength redshift can be ascribed to the increase in lattice distance caused by the swelling of PEGDA in water. These results demonstrate that the ZnS particles are movable in voids. We have prepared five photonic superstructures including one ZnS–silica/silica PC by co‐assembly strategy and four etched PCs from ZnS–silica/silica PC and ZnS–silica PC. It is difficult to fabricate these derived photonic superstructures using similar strategies from the conventional PCs based on silica or polystyrene particles.

Although brilliant colors have been achieved by conventional dip‐coating, drop‐casting, and fluidic cells approaches using ZnS^[^ ^49^, ^51^, ^55^, ^56^ ^]^ and CeO_2_^[^ ^46^ ^]^ particles as building blocks, the non‐close–assembling and the selective etching strategy has following advantages in: 1) fabricating non‐closely packed structures in a straightforward and efficient way; 2) avoiding the “coffee ring” effect; 3) tuning structural colors by simply altering *φ*_ZnS–silica_; 4) a wide tuning range of thicknesses; 5) constructing abundant PC derived superstructures; and 6) unique water‐responsive colors. These PCs and derived superstructures may advance the manipulation of light in different ways and may facilitate the applications of PCs in displays, sensing, anti‐counterfeiting, optical devices, and so on.

### 2.6 Information Encryption and Fluorescence Enhancement

**Figure 6:**
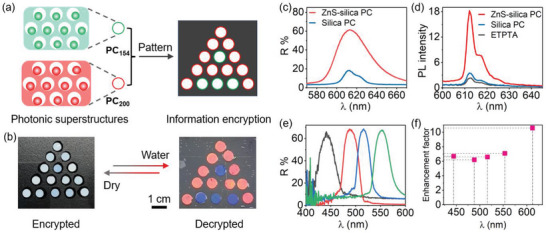
Photonic superstructures for information encryption and PCs for PL enhancement. a) Schematic illustration of the information encryption strategy based on photonic superstructures. b) Digital photos of the pattern under the dry and wetted states. c) Reflection spectra of the ZnS–silica and silica PCs. d) PL intensity of the Eu(TTA)_3_/ETPTA film covered on ZnS–silica PC, silica PC, and ETPTA films, respectively. e) Reflection of ZnS–silica PCs with different reflection wavelengths. f) Enhanced factor of the fluorescence when ZnS–silica PCs with different reflection wavelengths are used. The thicknesses of all PCs are fixed to 57 µm.

A new information encryption strategy is developed by taking the advantages of the unique “off–on” switch of reflection wavelengths of the water‐responsive photonic superstructures. Here, ZnS–silica PCs (*φ*_ZnS–silica_: 30%) with reflection wavelengths located at 606 and 472 nm (Figure S22, Supporting Information) have been prepared when ZnS–silica particles with size of 200 nm (core of 140 nm and shell of 30 nm) and 154 nm (core of 105 nm and shell of 24.5 nm) were used, respectively. After selective etching by HF, for easy discussion, corresponding HF‐etched PCs were named as PC_200_ and PC_154_, both of which show negligible reflectance (Figure S23, Supporting Information). Eleven PC_200_ are packed into “A” and four PC_154_ are packed as the background, thus forming a triangle pattern (**Figure** **6**a). As expected, under normal condition, this pattern shows white appearance and the “A” is encrypted and invisible (Figure 6b) due to the loss of ordered structures. In contrast, the red “A” with reflection wavelength at 632 nm (Figure S24, Supporting Information) can be decrypted from the blue background with a peak position located at 493 nm when this pattern is soaked in water because of recovery of long‐range order. The encryption–decryption process is highly reversible (Figure S25, Supporting Information). This encryption strategy with “off–on” color switch is different from conventional encryption methods based on the color switch from one to another, which will facilitate new applications in anti‐counterfeiting, displays, sensing, and so forth.

Except for information encryption, significant fluorescence enhancement can be realized when ZnS–silica PCs were used as substrates. For fluorescence enhancement, since the bandgap of the conventional silica PCs is narrow, the reflection peak position must be specifically altered to match the fluorescent wavelength, which could be inconvenient and difficult in practical applications. In addition, the low reflectance of conventional PCs could lead to the limited enhancement of fluorescence of dyes. In contrast, ZnS–silica PCs possess broad photonic bandgaps and high reflectance, which make them ideal candidates for enhancing fluorescence.

We design a double layered films with a fluorescent film covered on a PC to investigate the fluorescence enhancement. The freestanding fluorescent film was prepared by sandwiching the Eu(TTA)_3_/ETPTA solution (fluorescent peak: 612 nm) between two glasses, followed by a photopolymerization. ZnS–silica and silica PCs with reflection wavelengths located at 612 nm matching the fluorescent wavelength were used (Figure 6c). The thicknesses of the fluorescent and the PC films are ≈14 and ≈50 µm, respectively. When excited by the 405 nm, the fluorescent film shows a weak intensity on the ETPTA film (Figure 6d). Interestingly, the PL intensity is enhanced by a factor of ≈10 when the fluorescent film is covered on the ZnS–silica PC with reflection signal located at 612 nm. The fluorescence enhancement is mainly attributed to enhanced extraction of light generated in the PC, which can serve as the dielectric cavity and act as a local resonance mode for the emission propagation.^[^ ^57^, ^58^, ^59^, ^60^ ^]^ In contrast, there is only 1.7‐fold enhancement at the maximum emission wavelength of fluorescent film on the silica PC. The significant difference in enhancement can be attributed to the difference in reflectance between the ZnS–silica and silica PCs. In addition, the fluorescent intensity was enhanced by a factor of 6–7 when the reflection wavelengths of ZnS–silica PCs were located out of the emission of Eu(TTA)_3_ (Figure 6e,f), probably due to the scattering of light. Overall, the maximal enhancement of the fluorescence was obtained when the stopband overlaps the maximum emission wavelength of Eu(TTA)_3_.

## 3 Conclusion

In summary, bio‐inspired ZnS–silica PCs with intense reflectance, wide photonic bandgaps, highly saturated colors, long‐range order, and non‐closely packed structures were prepared by the non‐close–assembling of the uniform ZnS–silica core–shell particles with large *n* in ETPTA with a small *n*, followed by the photopolymerization. The silica shell enables the strong electrostatic repulsions between ZnS–silica particles and thus guarantees the highly ordered structures. Both the large Δ*n* and ordered structures are the major reasons for the outstanding optical performances of ZnS–silica PCs. The non‐close‐packing structure enables tunable brilliant structural colors covering most visible colors by simply altering *φ*_ZnS–silica_ of two ZnS–silica particles. These ZnS–silica PCs show intense reflectance with a wide range of thicknesses and a much smaller *T*_th_ than that of the PCs prepared with silica particles. A variety of new photonic superstructures were obtained by self‐assembling ZnS–silica and silica particles into ZnS–silica/silica PCs or by selective etching silica or ZnS of these PCs and the ZnS–silica PCs. Furthermore, a water‐responsive photonic superstructure was fabricated, which shows unique disorder–order switch and thus off–on structural colors under dry‐wetted states. Based on these specific properties, a new information encryption strategy is developed by simply combing these water‐responsive photonic superstructures. The information is encrypted under normal condition but decrypted in water and the encryption–decryption switch is highly reversible. It is shown that ZnS–silica PCs are excellent platform for enhancing fluorescence, showing ≈10‐fold enhancement by matching the reflection and fluorescent wavelengths, approximately six times higher than that of silica PC. This work not only offers direct non‐close‐packing–based brilliant structural colors but also develops derived PC superstructures, which open a new avenue to fabricate advanced photonic materials and may facilitate structural color related applications in displays, anti‐counterfeiting, and optical devices.

## 4 Experimental Section

### Materials

Silica particles (in ethanol, PDI < 0.03, *ζ*‐potential: −45 mV), tetraethyl orthosilicate (98%), ethanol (EtOH, 99%), and aqueous ammonia (28%) were purchased from Sinopharm Chemical Reagent Co. Ltd. ETPTA and 2‐hydroxy‐2‐methylpropiophenone (photo‐initiator, 96%) was obtained from Sigma‐Aldrich. Polyvinylpyrrolidone (PVP, Mw: 40 000), thioacetamide (TAA), and Zn(NO_3_)_2_ were purchased from Aladdin. All the chemicals were used as received without further purifications.

### Preparation of ZnS Particles

ZnS particles were synthesized according to the previous report.^[^ ^49^ ^]^ 142 nm ZnS colloids could be taken as the example. Briefly, PVP (10 g) was first dissolved into H_2_O (150 mL) to form a clear solution at room temperature. After being heated to 80 °C, HNO_3_ (0.2 mL) and TAA (7.135 g) were added to the PVP aqueous solution to form a transparent A solution. Then, Zn(NO_3_)_2_ (2.97 g) dissolved into H_2_O (50 mL) was added to the A solution and the final white solution was obtained after stirring for 2 h. The white ZnS particles were obtained after five times washing by excessive water. Here, 110 nm ZnS particles were obtained through similar fabrication procedures except reaction time was 1 h.

### Preparation of ZnS–Silica Core–Shell Particles

The as‐fabricated ZnS particles (0.8 g, 142 nm) were dispersed into the mixture of ethanol (160 mL), NH_4_OH (6 mL), and H_2_O (12 mL) by sonication. After vigorous stirring for 1 h, TEOS (3 mL) was added to the mixed solution. ZnS–silica was obtained after reacting for 12 h at 25 °C. The shell thickness can be controlled by altering the amount of TEOS. Here, the silica thickness was 15/21/50 nm when TEOS with 1/1.5/3 mL was used, respectively.

### Fabrication of ZnS–Silica PCs

The ZnS–silica particles (0.02–0.04 mL) were first dispersed into the mixture containing ETPTA (0.08–0.06 mL) and ethanol (0.5 mL) with a 5% photo‐initiator by sonication. Then, the mixed solution was heated to 100 °C in an oven and a liquid solution showing highly brilliant structural colors was obtained after 1 h. This liquid solution was sandwiched between two glasses with a thickness of 0.05 mm, which was then exposed to the UV light (365 nm, 4.8 mW cm^−2^) for 3 min to achieve the ZnS–silica PCs. The volume of the ZnS–silica plus that of ETPTA was kept constant as 0.1 mL.

### Fabrication of Derived Photonic Superstructures

The fabrication of ZnS–silica/silica PC was similar to that of ZnS–silica PC except both ZnS–silica and silica particles with the same volumes were used as the building blocks and ETPTA was replaced by PEGDA. Except for the ZnS–silica/silica PC, other derived photonic superstructures were prepared through selective etching the ZnS or silica of ZnS–silica/silica and ZnS–silica PCs. ZnS–silica PC could be taken as an example. One derived photonic structure was obtained by immersing the PC in HCl (10% wt) solution for 2 h and then washed by excessive water and dried. Another derived photonic superstructure was achieved by immersing the PC in HF (2% wt) for 20 min and then washed by excessive water and dried. Additional two derived photonic superstructures were fabricated through similar approaches except the ZnS–silica PC was replaced by the ZnS–silica/silica PC.

### Constructing Fluorescent Enhancement System

The Eu(TTA)_3_ (1 mg) was dissolved in ETPTA (1 mL) with the help of sonication. Then, 0.02 mL of the Eu(TTA)_3_/ETPTA was sandwiched between two glasses with an interval of 0.09 mm. Fluorescent film was obtained after exposing to UV light (365 nm, 4.8 mW cm^−2^) for 3 min. This film was covered on diverse substrates including ETPTA, silica PC, and ZnS–silica PC to test the fluorescent intensity of the Eu(TTA)_3_.

### Characterization

The structures of PCs were investigated by the SEM (HITACHI SEMSU8010). For most cases, the reflection spectra of PCs are obtained with the both incident light and collection angle fixed at 0°. The reflectance spectra were measured by a NOVA spectrometer (Hamamatsu, S7031). Angle‐resolved spectra were collected by simultaneously changing incident light and collection angles from 0 to 60°, using the angle‐resolved spectrum system (R1, Ideaoptics, China) equipped with a highly sensitive spectrometer (NOVA, Ideaoptics, China). Fluorescent tests were also conducted by fixing the indicant and reflective angles to 5° with a laser of 405 nm (400 mW) as a light source. The optical microscope images and microscopic reflectance spectra were obtained on an Olympus BXFM reflection‐type microscope operated in darkfield mode. The surface charge of the particles is measured by Malvern ZS 90. The simulated reflection spectra were achieved by commercial FDTD software (Lumerical. In simulations). The refractive indexes of ZnS and silica were set as 1.910 and 1.460, respectively. For PCs, a plane wave was placed above the ordered structure for a reflection spectral calculation

## Conflict of Interest

The authors declare no conflict of interest.

## Supporting information

Supporting Information

## Data Availability

Research data are not shared.
