# Supplementary material for: Bio‐Inspired Highly Brilliant Structural Colors and Derived Photonic Superstructures for Information Encryption and Fluorescence Enhancement
Source: Adv Sci (Weinh). 2023 Jun 17;10(24):2302240. doi: 10.1002/advs.202302240 (PMC10460858; doi:10.1002/advs.202302240)
Supplement: Supplementary file 1 — Supporting Information [file ADVS-10-2302240-s001.pdf]

ADVANCED SCIENCE
Open Access

# Supporting Information

for *Adv. Sci.*, DOI 10.1002/advs.202302240

Bio-Inspired Highly Brilliant Structural Colors and Derived Photonic Superstructures for Information Encryption and Fluorescence Enhancement

*Xiaoru Liu, Junfu Liu, Boru Wei, Dongpeng Yang*, Li Luo*, Dekun Ma and Shaoming Huang**

**Bio-inspired highly brilliant structural colors and derived photonic superstructures for information encryption and fluorescence enhancement**

Xiaoru Liu[a], Junfu Liu[a], Boru Wei[a], Dongpeng Yang*,[a], Li Luo*,[a], Dekun Ma[b], Shaoming Huang*,[a]

[a]School of Materials and Energy, School of Physics and Optoelectric Engineering, Guangzhou Key Laboratory of Low-Dimensional Materials and Energy Storage Devices, Guangdong University of Technology, Guangzhou 510006, P. R. China.
[b]Zhejiang Key Laboratory of Alternative Technologies for Fine Chemicals Process, Shaoxing University, Shaoxing 312000, P. R. China.

E-mail: dpyang@gdut.edu.cn (D. P. Yang); luoli@gdut.edu.cn (L. Luo); smhuang@gdut.edu.cn (S. M. Huang)

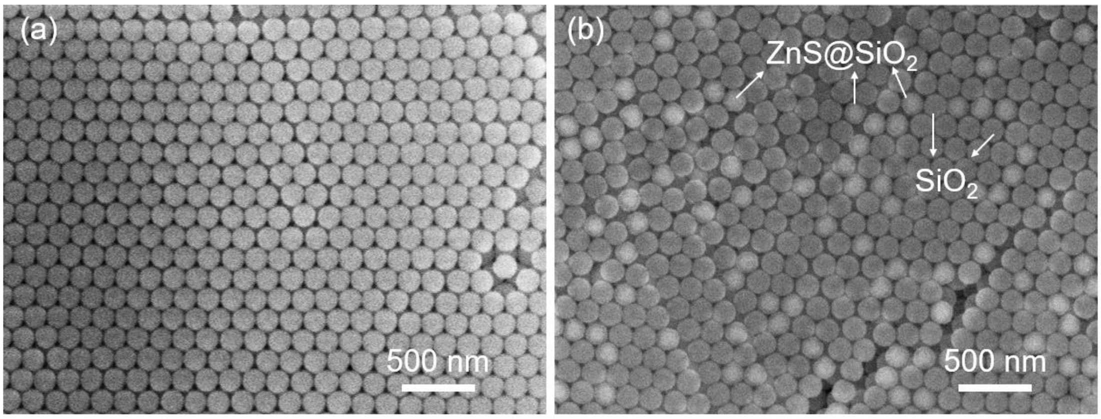


Figure S1. SEM image of (a) silica particles and (b) the mixture of ZnS-silica and silica particles.

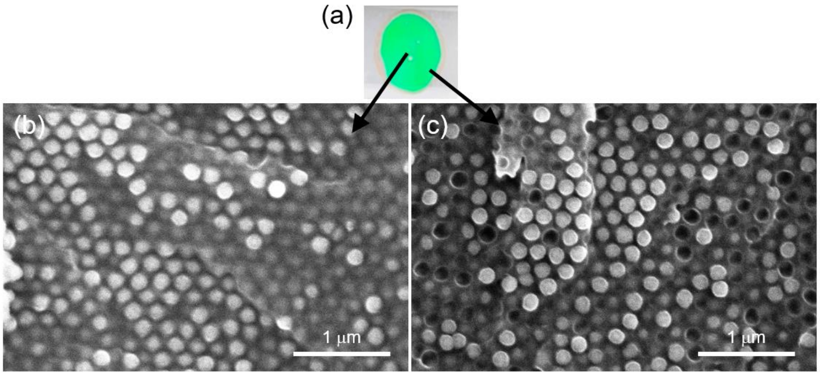


Figure S2. (a) Digital photo of the ZnS-silica PC fabricated with 158 nm ZnS (core: 110 nm and shell: 24 nm) and with $\varphi_{ZnS\text{-}silica}$ of 20%. The cross-sectional SEM images of the (b) central and (c) rim regions of the ZnS-silica PC.

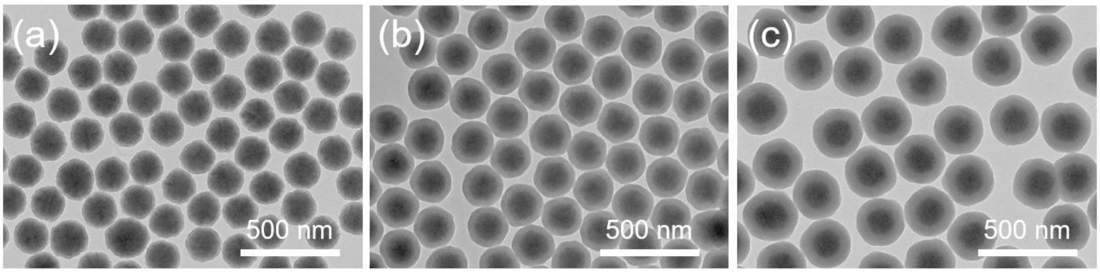


Figure S3. TEM images of ZnS-silica particles with different sizes: (a) 178 nm (core: 142 and shell: 18 nm), (b) 212 nm (core: 142 and shell: 35 nm), and (c) 248 nm (core: 142 and shell: 53 nm).

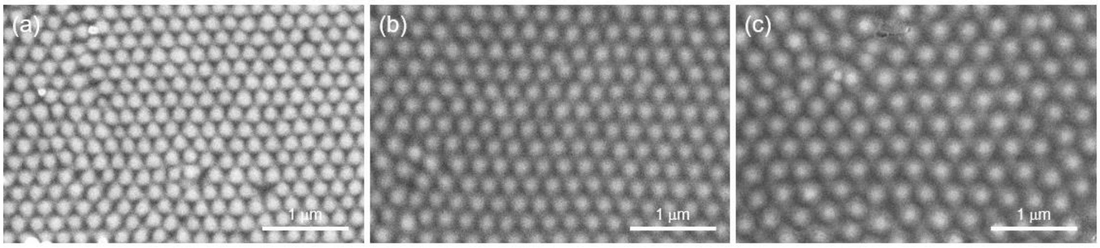


Figure S4. SEM images of ZnS-silica PCs with the same ZnS core (142 nm) but different silica shell thicknesses: (a) 18 nm, (b) 35 nm, and (c) 53 nm.

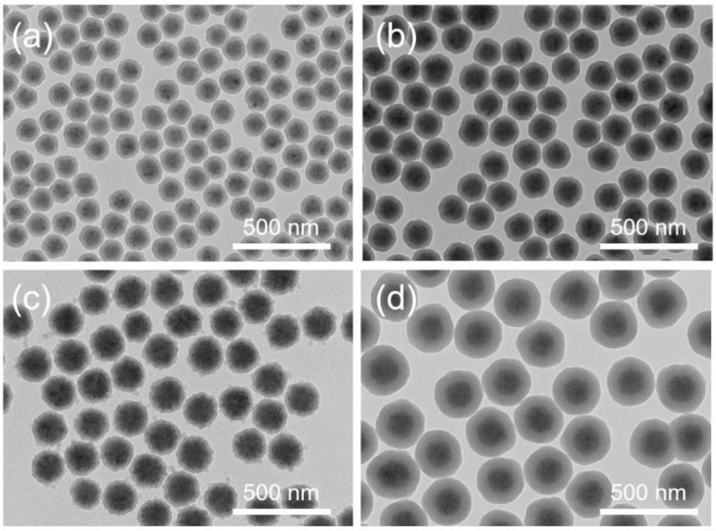


Figure S5. TEM images of ZnS-silica particles with different sizes: (a) 128 nm (core: 92 and shell: 18 nm), (b) 158 nm (core: 110 and shell: 24 nm), (c) 184 nm (core: 142 and shell: 21 nm), and (d) 248 nm (core: 142 and shell: 53 nm).

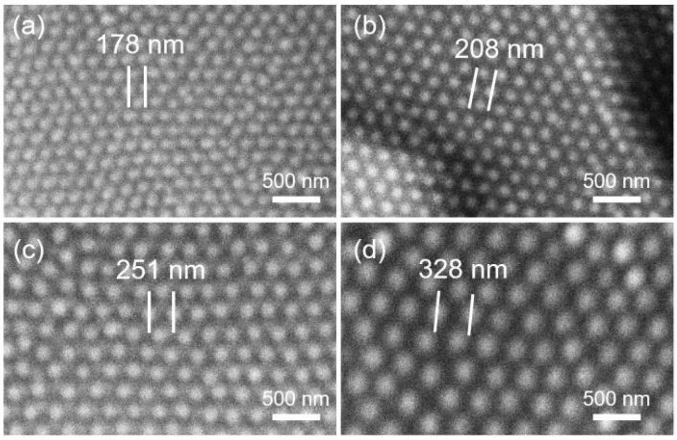


Figure S6. SEM images of ZnS-silica PCs ($\varphi_{ZnS\text{-}silica}$ fixed to 20%) particle sizes of (a) 128, (b) 158, (c) 184, and (d) 248 nm.

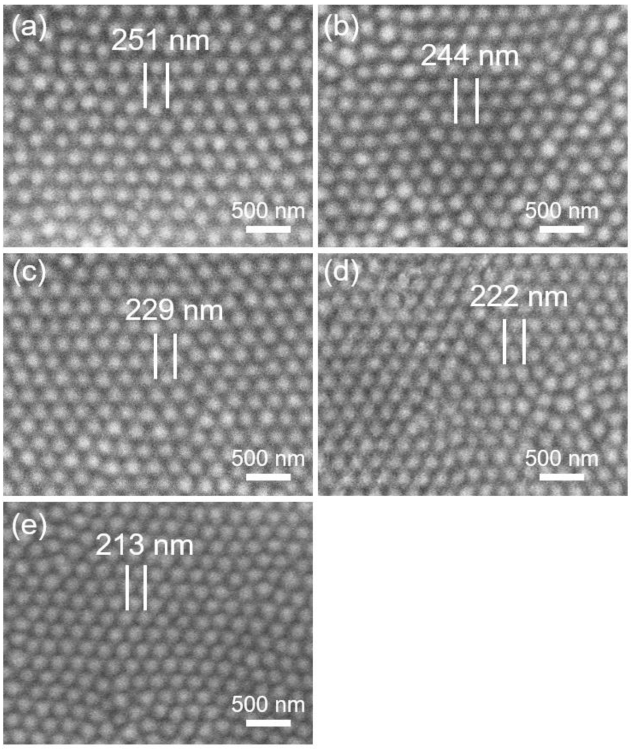


Figure S7. SEM images of ZnS-silica (184 nm) PCs with $\varphi_{ZnS\text{-}silica}$ of (a) 20%, (b) 25%, (c) 30%, (d) 35%, and (e) 40%.

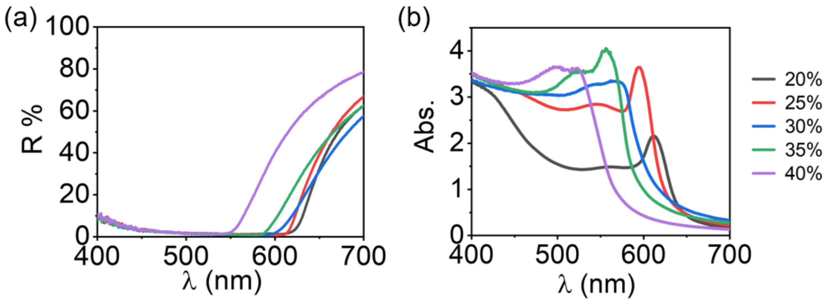


Figure S8. (a) Transmittance and (b) absorbance spectra of the PCs with different $\varphi_{ZnS\text{-}silica}$ (20-40%). ZnS-silica particle: 184 nm (core: 142 nm and shell: 21 nm).

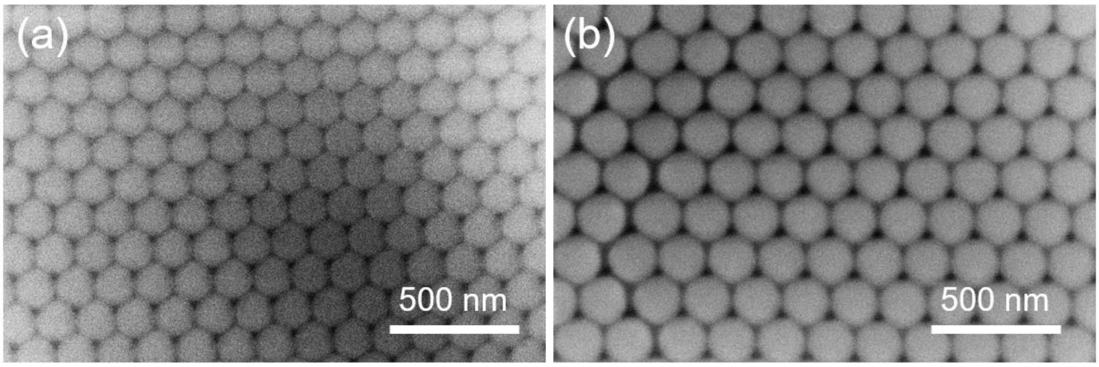


Figure S9. SEM images of silica particles with the size of (a) 160 nm and (b) 186 nm.

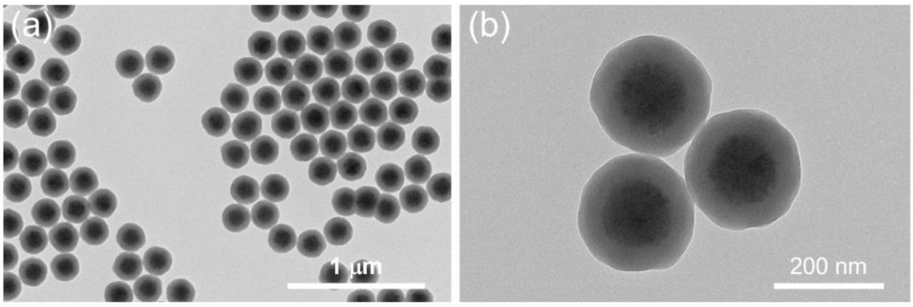


Figure S10. TEM images of ZnS-silica particles with the core of 120 nm and shell of 45 nm.

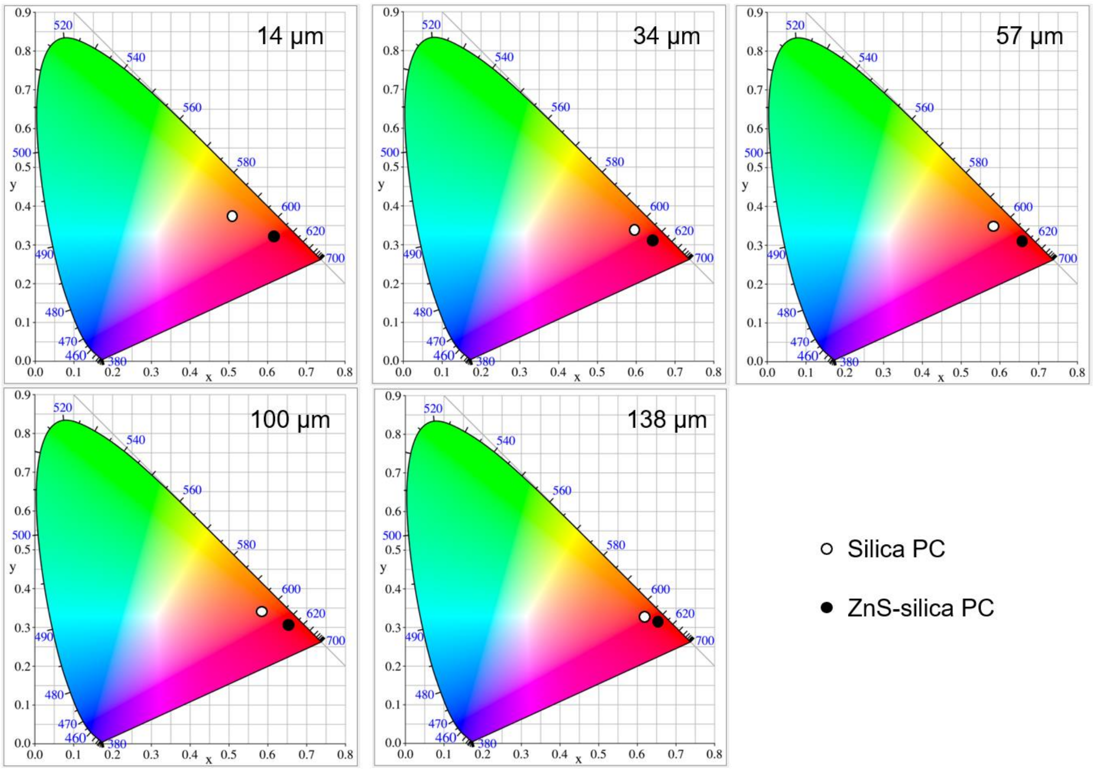


Figure S11. CIE diagram of silica and ZnS-silica PCs with different thicknesses (14-138 μm).

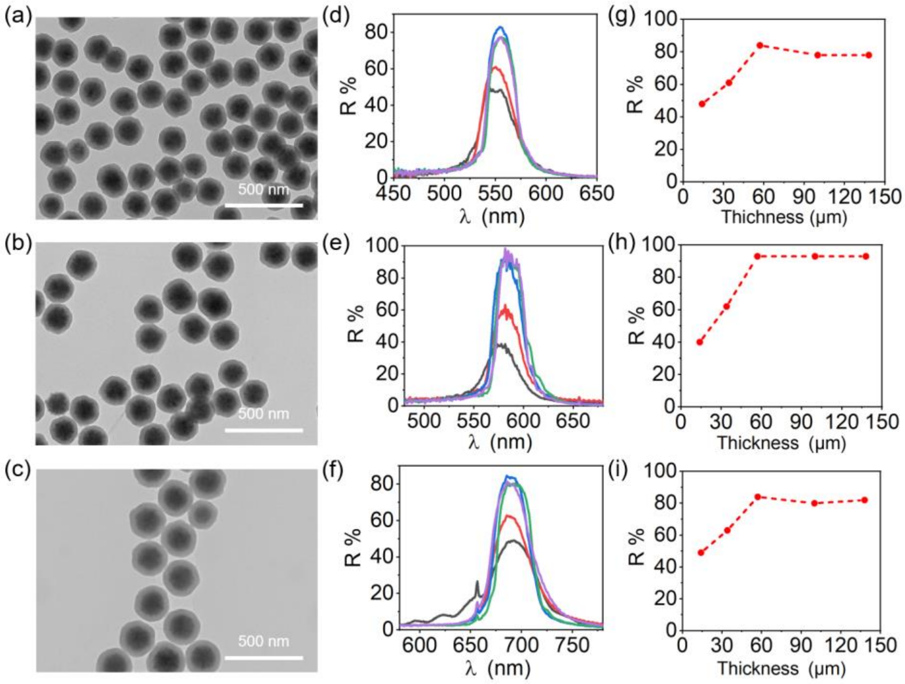


Figure S12. (a-c) TEM images of ZnS-silica particles, (d-f) reflection spectra of ZnS-silica PCs, and corresponding (g-i) reflectance as a function film thickness based on the same silica shell thickness (30 nm) and $\varphi_{ZnS\text{-}silica}$ (35%) but different core size: (a, d) 127 nm, (b, e) 140 nm, and (c, f) 152 nm.

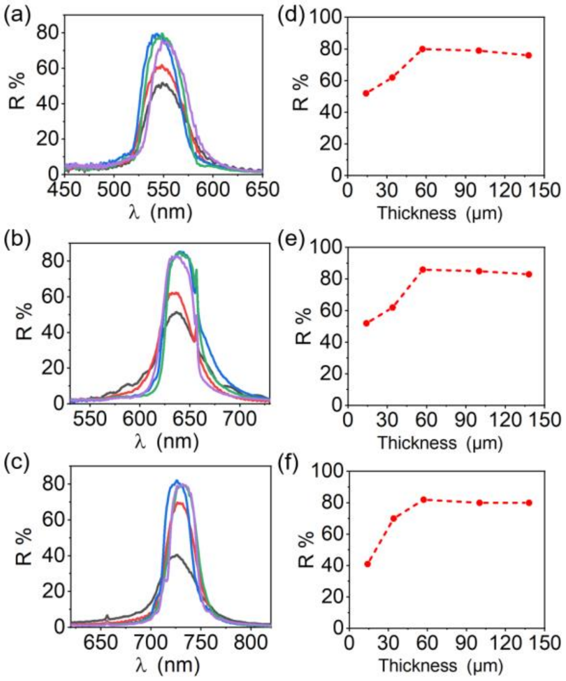


Figure S13. (a-c) Reflection spectra of ZnS-silica PCs and corresponding (d-f) reflectance as a function film thickness based on the same ZnS core (142 nm) and $\varphi_{ZnS\text{-}silica}$ (35%) but different silica shell thicknesses: (a, d) 18 nm, (b, e) 35 nm, and (c, f) 53 nm. The TEM images of these ZnS-silica particles can be found in Figure S2.

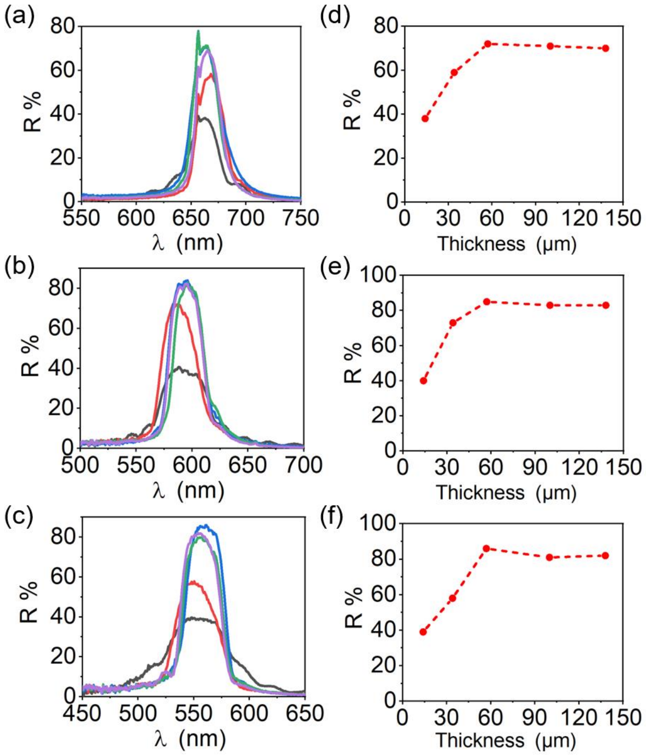


Figure S14. (a-c) Reflection spectra of ZnS-silica PCs and corresponding (d-f) reflectance as a function film thickness based on the same ZnS-silica particle (core of 140 nm and shell of 30 nm) but different $\varphi_{ZnS\text{-}silica}$ (35%): (a, d) 20%, (b, e) 30%, and (c, f) 40%. The TEM images of the ZnS-silica particles can be found in Figure S9b.

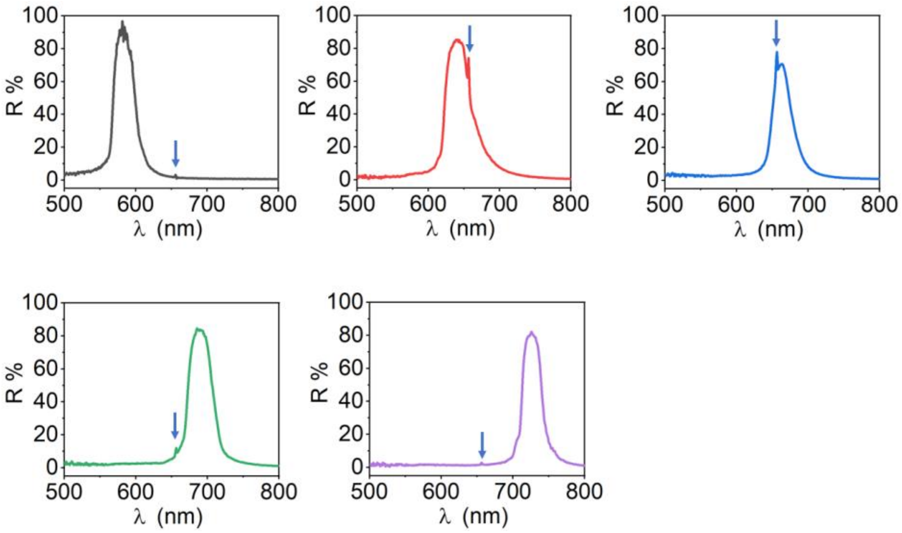


Figure S15. Reflection spectra of ZnS-silica PCs with reflection wavelengths located at (a) 581, (b) 640, (c) 663, (d) 689, and (e) 726 nm.

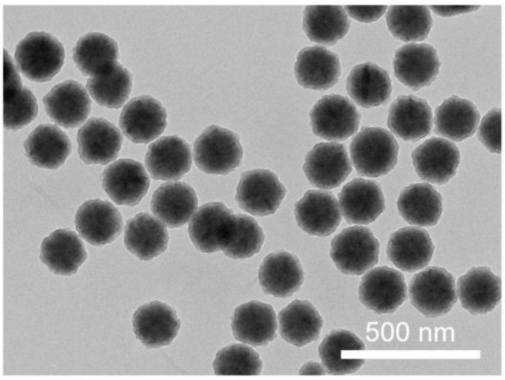


Figure S16. TEM image of ZnS-silica particles with the core of 142 nm and shell of 15 nm.

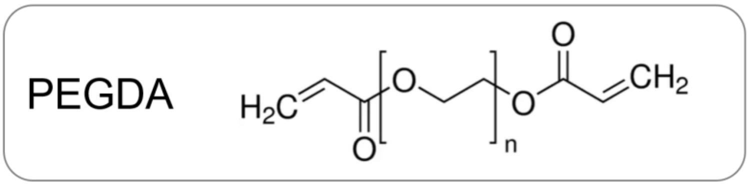


Figure S17. Chemical structure of poly (ethylene glycol) diacrylate (PEGDA).

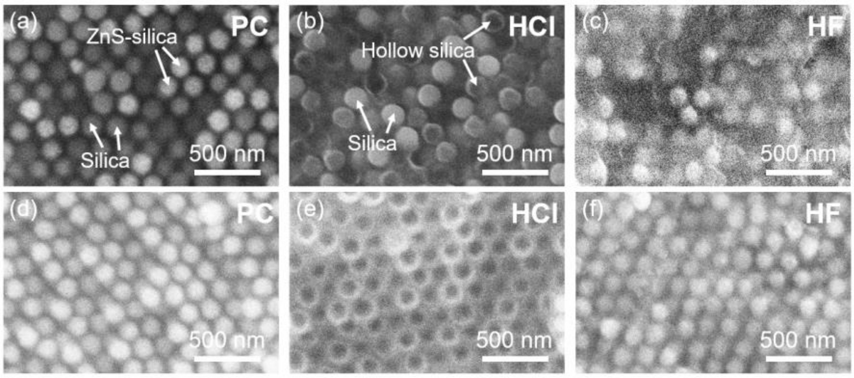


Figure S18. Cross-sectional SEM images of (a) ZnS-silica/silica PC, (b) corresponding HCl etched PC, and (c) HF etched PC. Cross-sectional SEM images of (d) ZnS-silica PC, (e) corresponding HCl etched PC, and (f) HF etched PC.

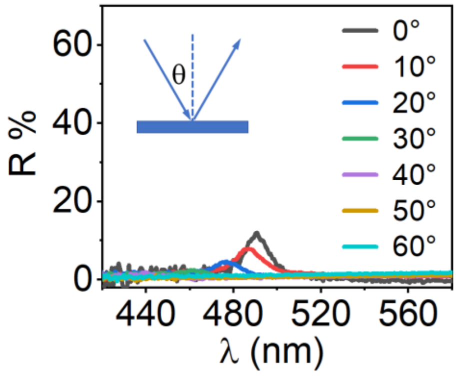


Figure S19. Angle-resolved spectra of the ZnS-silica/silica PC etched by HF.

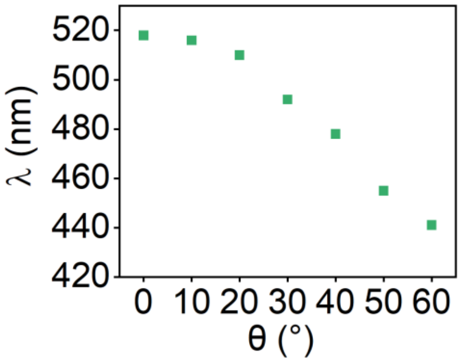


Figure S20. Angle-resolved spectra of the ZnS-silica PC etched by HF.

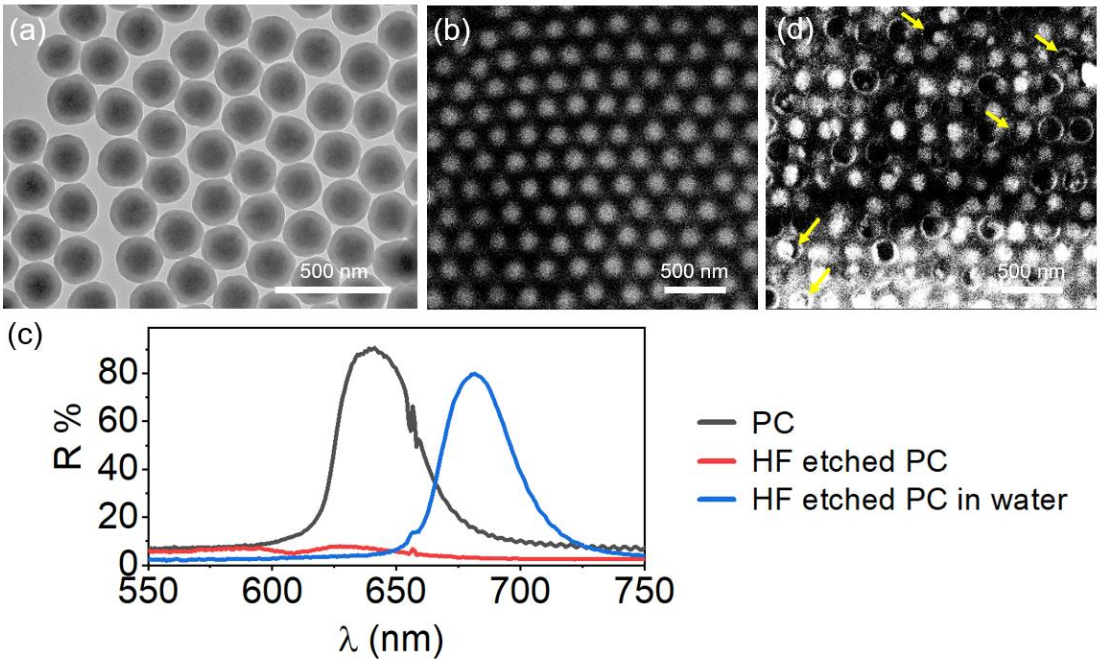


Figure S21. (a) TEM image of ZnS-silica particles. (b) The cross-sectional SEM image of the pristine ZnS-silica PC. (c) Reflection spectra of the ZnS-silica PC under different states. (d) Cross-sectional SEM image of the ZnS-silica PC after being etched by HF. ZnS-silica particle: core of 142 nm and shell of 35 nm.

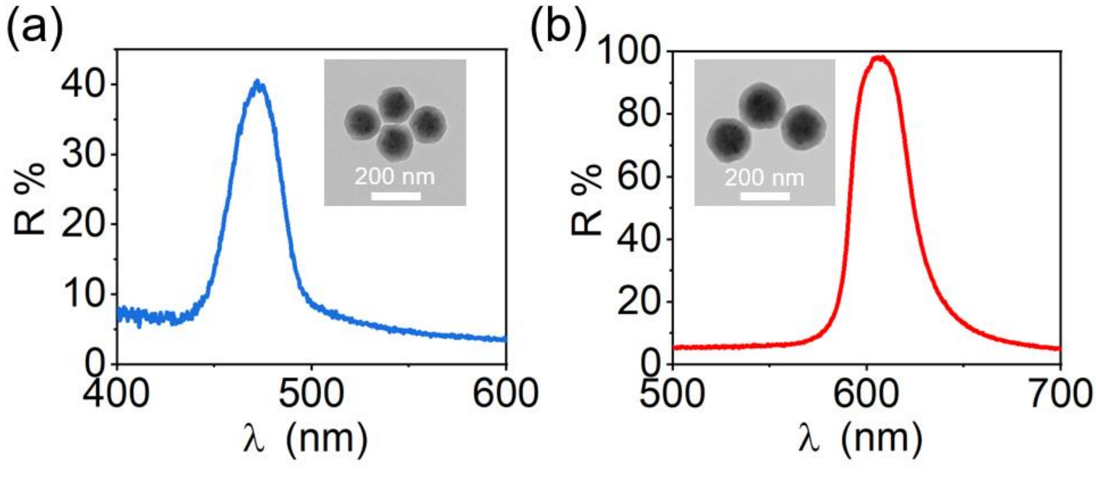


Figure S22. Reflection spectra of ZnS-silica PCs and corresponding TEM images of ZnS-silica particles with the particle size of (a) 200 nm (the core of 140 nm and shell of 30 nm) and (b) 154 nm (the core of 105 nm and shell of 24.5 nm). The $\varphi_{ZnS\text{-}silica}$ of both ZnS-silica PCs is 30%.

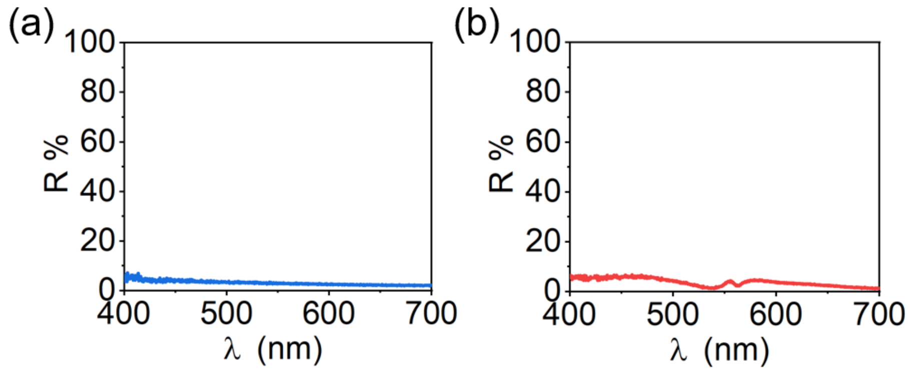


Figure S23. Reflection spectra of HF-etched ZnS-silica PCs, named (a) $PC_{154}$ and (b) $PC_{200}$.

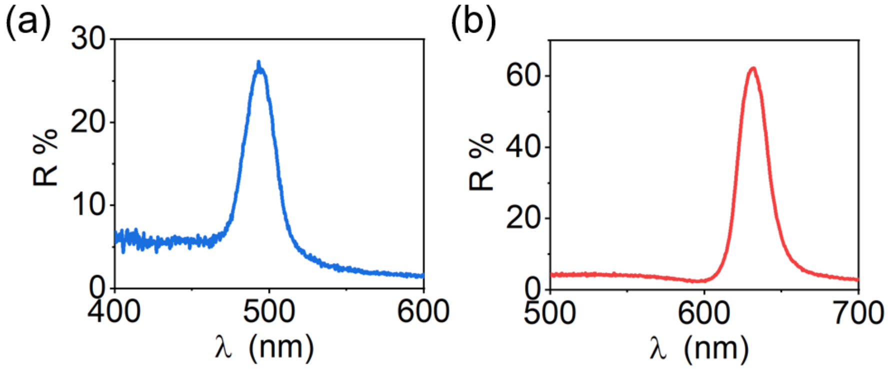


Figure S24. Reflection spectra of (a) $PC_{154}$ and (b) $PC_{200}$ immersed in water.

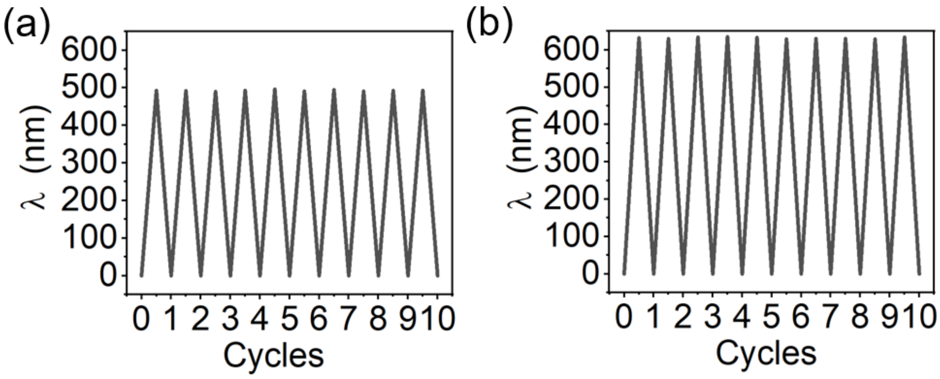


Figure S25. Reflection wavelengths of (a) $PC_{154}$ and (b) $PC_{200}$ as the function of the dry-wetted cycle.

Table S1. Comparison of the measured and calculated reflection wavelengths (λ) of ZnS-silica PCs with different $\varphi_{ZnS\text{-}silica}$ (20-40%) and with a particle size of 184 nm.

| $\varphi_{ZnS\text{-}silica}$ | Measured λ (nm) | Calculated λ (nm) |
|---|---|---|
| 20% | 617 | 621 |
| 25% | 601 | 608 |
| 30% | 575 | 575 |
| 35% | 561 | 562 |
| 40% | 538 | 545 |

Table S2. Comparison of the measured and calculated reflection wavelengths (λ) of ZnS-silica PCs with a fixed $\varphi_{ZnS\text{-}silica}$ of 20% but different particle sizes (128-248 nm).

| Size (nm) | Measured λ (nm) | Calculated λ (nm) |
|---|---|---|
| 128 | 430 | 436 |
| 158 | 530 | 509 |
| 184 | 617 | 621 |
| 248 | 825 | 798 |
